# Role of Fibroblast Populations in Periodontal Wound Healing and Tissue Remodeling

**DOI:** 10.3389/fphys.2019.00270

**Published:** 2019-04-24

**Authors:** Patricio C. Smith, Constanza Martínez, Jorge Martínez, Christopher A. McCulloch

**Affiliations:** ^1^ Faculty of Medicine, School of Dentistry, Pontificia Universidad Católica de Chile, Santiago, Chile; ^2^ Laboratory of Cell Biology, Institute of Nutrition and Food Technology, INTA, Universidad de Chile, Santiago, Chile; ^3^ Faculty of Dentistry, University of Toronto, Toronto, ON, Canada

**Keywords:** periodontal, gingival, wound healing, connective tissue, fibroblast

## Abstract

After injury to periodontal tissues, a sequentially phased healing response is initiated that enables wound closure and partial restoration of tissue structure and function. Wound closure in periodontal tissues involves the tightly regulated coordination of resident cells in epithelial and connective tissue compartments. Multiple cell populations in these compartments synergize their metabolic activities to reestablish a mucosal seal that involves the underlying periodontal connective tissues and the attachment of these tissues to the tooth surface. The formation of an impermeable seal around the circumference of the tooth is of particular significance in oral health since colonization of tooth surfaces by pathogenic biofilms promotes inflammation, which can contribute to periodontal tissue degradation and tooth loss. The reformation of periodontal tissue structures in the healing response centrally involves fibroblasts, which synthesize and organize the collagen fibers that link alveolar bone and gingiva to the cementum covering the tooth root. The synthesis and remodeling of nascent collagen matrices are of fundamental importance for the reestablishment of a functional periodontium and are mediated by diverse, multi-functional fibroblast populations that reside within the connective tissues of gingiva and periodontal ligament. Notably, after gingival wounding, a fibroblast sub-type (myofibroblast) arises, which is centrally involved in collagen synthesis and fibrillar remodeling. While myofibroblasts are not usually seen in healthy, mature connective tissues, their formation is enhanced by wound-healing cytokines. The formation of myofibroblasts is also modulated by the stiffness of the extracellular matrix, which is mechanosensed by resident precursor cells in the gingival connective tissue microenvironment. Here, we consider the cellular origins and the factors that control the differentiation and matrix remodeling functions of periodontal fibroblasts. An improved understanding of the regulation and function of periodontal fibroblasts will be critical for the development of new therapies to optimize the restoration of periodontal structure and function after wounding.

## Wound Healing in the Periodontium: An Overview

**Figure 1:**
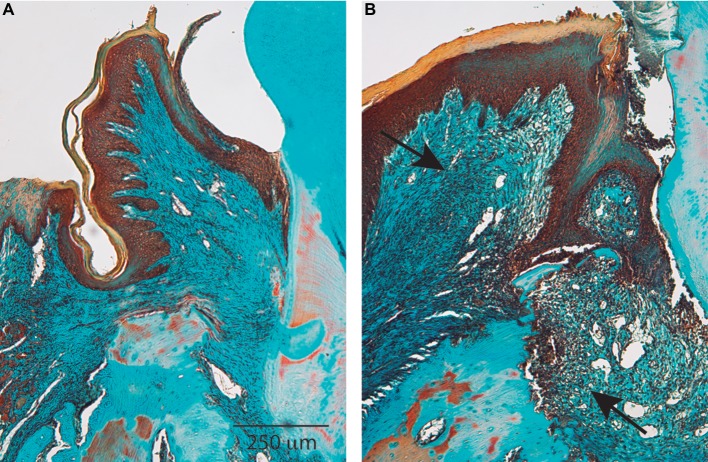
Healthy and diseased periodontium. Histological sections show the most significant characteristics of periodontal tissues in health **(A)** and disease **(B)**. Periodontal disease is characterized by chronic nonresolving inflammation in which the wound healing response is not able to regenerate tissues. Arrows indicate the tissue area affected by chronic inflammation. Magnification bar = 250 μm.

In metazoans, wound healing comprises a series of sequential phases that are initiated after tissue damage. In certain animal species, such as salamanders, wound healing can lead to the complete regeneration of the original tissues (e.g., leg or tail) after amputation. But in many mammalian species, wound healing leads to repair phenomena in which the original form and function of the tissue are not reestablished. In periodontal tissues of mammals that have been diminished by disease, complete tissue regeneration after wound healing is not achieved. Instead, large variations of reparative responses leading to scarring and inadequate tissue formation are frequently observed. Notably, if wound infection is present, as frequently occurs in healing periodontium, or in tissues affected by periodontitis (Pacios et al., 2012) (Figure 1), or in certain ulcerative disorders affecting the oral mucosae, a chronic healing response develops in which wounds do not close. Disorders of wound healing and the development of nonhealing wounds are exacerbated in vulnerable individuals including diabetics and older adults (Jones et al., 2018). Further, if pro-inflammatory mediators are continuously elevated, as is seen in periodontitis, idiopathic pulmonary lung disease, and glomerulonephritis, there is often extensive degradation followed by excessive production of disorganized connective tissue matrices. The formation of these dense, dysfunctional, highly cross-linked collagen matrices is frequently manifested in fibrotic lesions that affect a large array of organs and tissues, including the periodontium (Coelho and McCulloch, 2016). In view of the high prevalence of periodontal diseases in Western societies and the morbidity associated with the diseases and their treatment, it is of interest to obtain an improved understanding of the role of connective tissue cells in normal and delayed periodontal wound healing.

A large cadre of different cell types participates in the wound healing response. In the periodontium, some of the critically important cells include mesenchymal progenitor cells, osteoblasts, neutrophils, monocytes and macrophages, lymphocytes, dendritic cells, endothelial cells, keratinocytes, and fibroblasts (Chrysanthopoulou et al., 2014). Following wounding, resident tissue cells are activated through multiple signaling systems that promote increased gene expression, which in turn affect cell proliferation, differentiation, and the migration of precursor cells into the wound environment (Gurtner et al., 2008). The migration of matrix-synthesizing and matrix-remodeling cells into the wound strongly affects healing outcomes since the reformation of the extracellular matrix is critically important for the restoration of tissue form and function (Hinz, 2016).

During early phases of wound healing, a provisional matrix composed primarily of platelets, fibrin, and fibronectin, undergoes progressive, time-dependent alterations of organization, composition, and structure. These alterations involve the sequential degradation of the original components of the nascent matrix and their replacement by newly synthesized matrix molecules including collagens, fibronectin and proteoglycans (Rognoni et al., 2018). Matrix remodeling is critically important for optimized cell migration, tissue repopulation, and cell differentiation, processes that ultimately are essential for restoration of tissues damaged by disease or trauma. In addition, matrix stiffness, which is mechanosensed by cells as they apply actomyosin-mediated contractile forces to fibrillar collagen, strongly influences the outcomes of wound healing. As cells migrate into wounds, they sample the biophysical properties of the pericellular matrix. These sampling activities provide an important source of information for cells that contributes to the regulation of gene expression and myriad other cellular functions (Rognoni et al., 2018). In sum, the healing of connective tissues in general and the periodontium in particular requires the activities of resident cell populations that can synthesize structurally appropriate extracellular matrices, which in turn can support cell activation, proliferation, and differentiation.

The wound healing response involves three temporally overlapping yet distinct phases: (1) blood coagulation and inflammation; (2) nascent tissue formation; and (3) tissue remodeling (Gurtner et al., 2008). In the following sections, we consider briefly these first two phases of wound healing. Then, later on in this article, we will focus in particular on phase (3), tissue remodeling.

### Blood Coagulation and Inflammation Phase

Almost immediately after injury to periodontal tissues, the blood coagulation cascade is activated to control local arteriolar and venular bleeding. A platelet plug is formed that includes a mixture of fibrin and fibronectin, which collectively provide the initial mechanical support and scaffolding that is needed for tissue healing to occur. From their granules, locally activated platelets release growth factors, cytokines, and chemokines that stimulate cell proliferation, adhesion, and migration (Dovi et al., 2003).

Neutrophils are key cells that enter into the wound within hours after injury to phagocytose and eliminate contaminating microorganisms. These cells are recruited by soluble factors released by platelets, components derived from the complement cascade, and from bacteria (Kolaczkowska and Kubes, 2013). The number of neutrophils populating the wound is usually maximal at 2 days after wounding and their numbers decrease subsequently in the absence of overt infection.

Although engaged somewhat later in the wound healing process, tissue macrophages, which differentiate from circulating monocytes, migrate into the wound and play an important role in immune regulation and the release of growth factors that are needed to initiate the proliferation and migration of connective tissue cells of the periodontium (Davies et al., 2013). At least two different phenotypes of macrophages have been identified during wound healing. Although the exact nature of these phenotypes is currently provisional and as their nomenclature is being reconsidered, we note that historically, macrophages have been grouped into M1 (inflammatory) or M2 (alternatively activated or reparative macrophage) subpopulations. These cells are sequentially recruited into the wound at the beginning and end of the inflammatory phase, respectively (Novak and Koh, 2013). Notably, a relative paucity of the M2 phenotype has been associated with delayed wound healing (Klinkert et al., 2017).

### Nascent Tissue Formation

The phase during which new tissue is formed occurs ~2–10 days after injury (Gurtner et al., 2008) and involves the proliferation and migration of epithelial, connective tissue, and endothelial cells. In the context of mucosal sealing to prevent microbial colonization of deeper connective tissues, a central feature of this phase of wound healing is the proliferation and migration of epithelial cells over the surface of the wound and around the tooth circumference (Gurtner et al., 2008). Increased proliferation of cells in the basal layers of the oral, sulcular, and junctional epithelia is particularly marked at 48–72 h after injury (Odland and Ross, 1968). The interactions of migrating keratinocytes with underlying matrix molecules including type I collagen, fibronectin, and polymerized fibrin are critical for the repopulation of the oral epithelia of the dentogingival junction (Sudbeck et al., 1997). The increased avidity and affinity of epithelial cell integrins, which is regulated by allosteric mechanisms in these migrating cells, is functionally linked to the increased expression and activation of matrix metalloproteinases that degrade collagen and enhance the directional migration of keratinocytes (Dumin et al., 2001).

The formation of new blood vessels is required for metabolic perfusion and the subsequent growth of new cells that repopulate the wound site. Several soluble factors released by platelets and local macrophage populations stimulate angiogenesis (Dor et al., 2003). New blood vessels can emerge from pre-existing capillaries by the proliferation of endothelial precursor cells and may also involve, to a limited extent, the differentiation of circulating monocytes into endothelial cells (Dor et al., 2003). Newly formed blood vessels form, sprout and invade the nascent wound healing matrix, a process that is accompanied by the formation of perivascular fibroblasts and by an increased number of tissue macrophages. Together these cells contribute to the replacement of the temporary fibrin matrix with a more mature and stiffer provisional matrix that contains more fibronectin and polymeric collagen than the earlier phases of wound healing.

Connective tissue fibroblasts are critically involved in the wound healing response as a result of their contributions to the formation of new tissues and subsequent tissue remodeling. As this review is focused specifically on the role of connective tissue fibroblasts in periodontal wound healing and tissue remodeling, these themes will be developed in more detail below.

## Healing of Periodontal Connective Tissues

**Figure 2:**
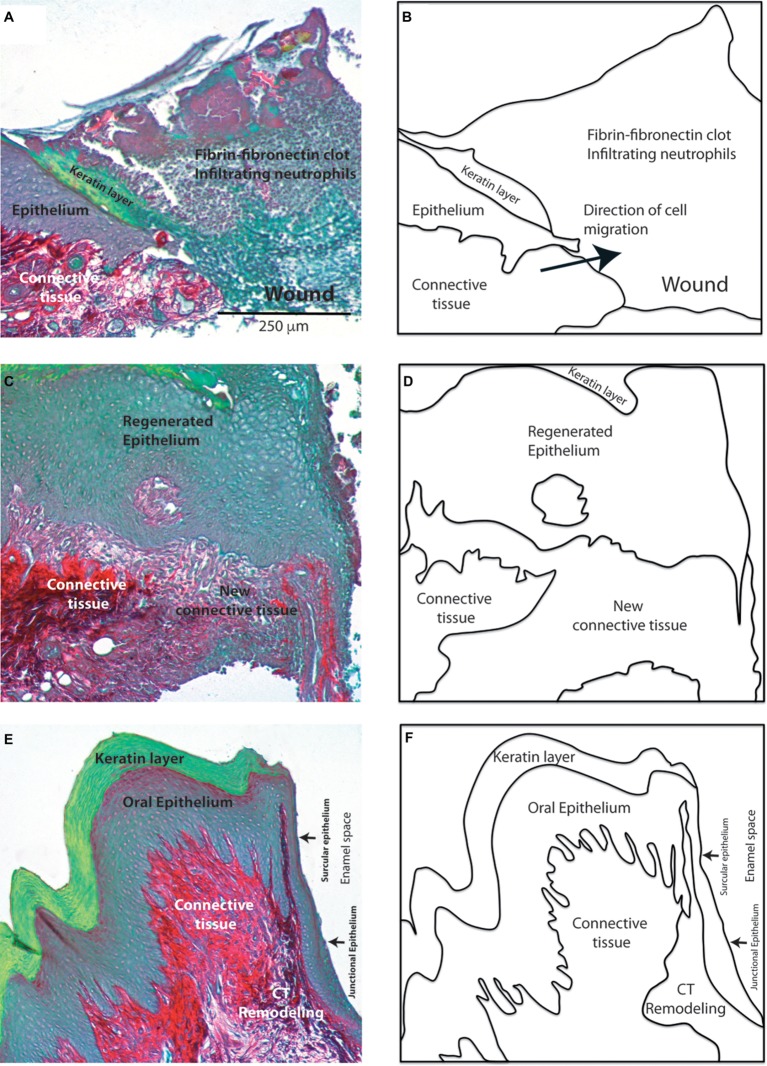
Morphological features of periodontal wound healing. Histological sections stained with sirius red show the collagen network (red). Images show the main histological changes in 2 **(A)**, 7 **(C)** and 15 **(E)** day old gingival wounds. Diagrams in **(B)**, **(D)**, and **(F)** show the main tissues involved in these responses. Magnification bar = 250 μm.

One critical aspect of the wound healing response in the periodontium is the regeneration of the collagen fibers that connect the cementum covering the root of the tooth to gingival connective tissues and the alveolar bone. The main histological features that are observed during periodontal wound healing highlight the critical role of collagen-synthesizing cells in the restoration of periodontal connective tissue structure and function (Figure 2). Importantly, fibroblasts in the lamina propria of gingival connective tissues secrete and organize discrete collagen networks. These networks connect the cementum overlying the root surface with the gingival lamina propria and the alveolar bone with the lamina propria.

Connective tissue fibroblasts play a central role in the new tissue formation and remodeling phases of wound healing. Although the biological phenomena associated with these two phases are observed in all types of wounded tissues, the duration of these phases may vary according to multiple factors. These factors include the location and size of the wound, the tissue vascularity, the abundance of potentially repopulating cells, and the functional role of the tissue itself. In general, wounds in which the wound edges are closely approximated (so-called “primary intention”) heal relatively quickly, whereas wounds in which the edges are not approximated (“secondary intention”) exhibit slower healing. In these types of wounds, ingrowth of granulation tissue into the denuded space is required prior to recolonization by epithelial cells.

**Figure 3:**
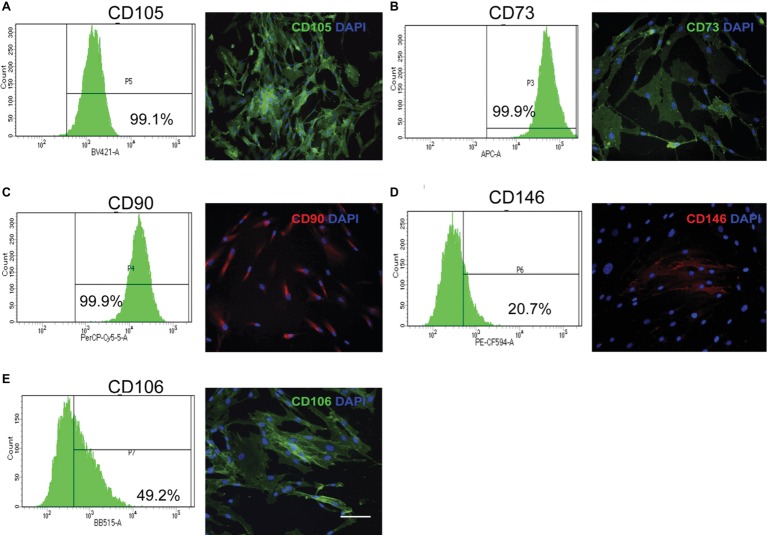
Stem cell mesenchymal markers in primary cultures of human periodontal connective tissue cells. Human periodontal ligament cells were immunostained for selected cell surface markers, counterstained with DAPI, and evaluated through flow cytometry, using an antibody panel. Flow cytometry histograms with percentage of positive cells and representative immunofluorescence images are shown. **(A)** CD105 **(B)** CD73 **(C)** CD90 **(D)** CD146 **(E)** CD106. Magnification bar equals 50 μm. (Images in this figure correspond to reanalysis of previously published data in Martinez et al., 2016).

Within 30 h after wounding of the periodontium, connective tissue fibroblasts exit the G_0_ phase of the cell cycle and enter the proliferative phase of the cell cycle to contribute to cell repopulation of these tissues (Gould et al., 1980). Most of the fibroblast progenitors that contribute to wound repopulation and maintenance of the tissue steady state arise from cell populations that are located adjacent to small blood vessels and are distributed throughout the periodontium (McCulloch and Melcher, 1983). These periodontally derived mesenchymal progenitor cells exhibit similar phenotypes as bone marrow stromal-derived mesenchymal progenitor cells, which is shown by their extensive self-renewal capacity and by their ability to differentiate into multiple cell linages as determined both *in vitro* and *in vivo* (Fournier et al., 2010; Fournier et al., 2016). Some of the phenotypic markers that have been detected in these progenitor cell populations include STRO-1, CD105, CD73, CD90; CD146, CD106, SSEA-4, CD271, Nanog, Sox-2, and Oct-4 (Tang et al., 2011; Jin et al., 2015). The most relevant cell surface markers detected in human periodontal ligament progenitor cells are shown in Figure 3. Notably, and consistent with much earlier morphological studies of tooth formation (Ten Cate et al., 1971), periodontal progenitor cells appear to arise from a neural crest origin (Tang et al., 2011; Xu et al., 2013). Mesenchymal progenitor cells isolated from human gingiva also display neural crest-related markers and exhibit the ability to generate progeny with the capacity to differentiate along the neural crest lineage. These findings suggest that gingival tissues contain progenitor cells with a wide differentiation repertoire that could potentially be harnessed for diverse tissue regenerative approaches (Xu et al., 2013; Fournier et al., 2016).

In addition to local resident fibroblast progenitor cells, a circulating cell population derived from bone marrow (known as fibrocytes) may also migrate into wounds and contribute to tissue regeneration (Kao et al., 2011). Currently, the biological significance of circulating fibrocytes in the context of wound healing in general and periodontal regeneration in particular is not known although earlier data using parabiotic methods (Ross et al., 1970) and radiotracer techniques (Gould et al., 1980) showed that circulating cells make only a very small contribution to cell repopulation of healing wounds.

The activation of connective tissue fibroblasts in response to wound healing is driven by soluble mediators that include chemokines, cytokines, and growth factors. These molecules stimulate cell proliferation, migration, differentiation, and the control of matrix protein synthesis and degradation (Iyer et al., 1999). In addition to these soluble factors, connective tissue cell responses are affected by modifications in the mechanical stiffness that are sensed by cell matrix adhesions. For example, human gingival fibroblasts will differentiate into myofibroblasts in response to TGF-b1 but only if cultured on stiff (but not soft) collagen substrates (Arora et al., 1999). These data demonstrate that matrix stiffness plays a central role in the cell signaling that regulates central features of fibroblast behavior during wound healing.

During the remodeling phase of wound healing, a specific sub-type of fibroblast may emerge. These cells (myofibroblasts) (Gabbiani et al., 1978) are actively engaged in the secretion and remodeling of the wound matrix in cells from periodontal tissues (Giannopoulou and Cimasoni, 1996; Smith and Martínez, 2006). Several cell types may contribute to the formation of myofibroblasts, and the origin of these cells in wounded periodontal tissues is not well-defined. Myofibroblasts may derive from connective tissue fibroblasts surrounding the wound, mesenchymal stem cells, pericytes, circulating fibrocytes derived from the bone marrow, and epithelial cells.

**Figure 4:**
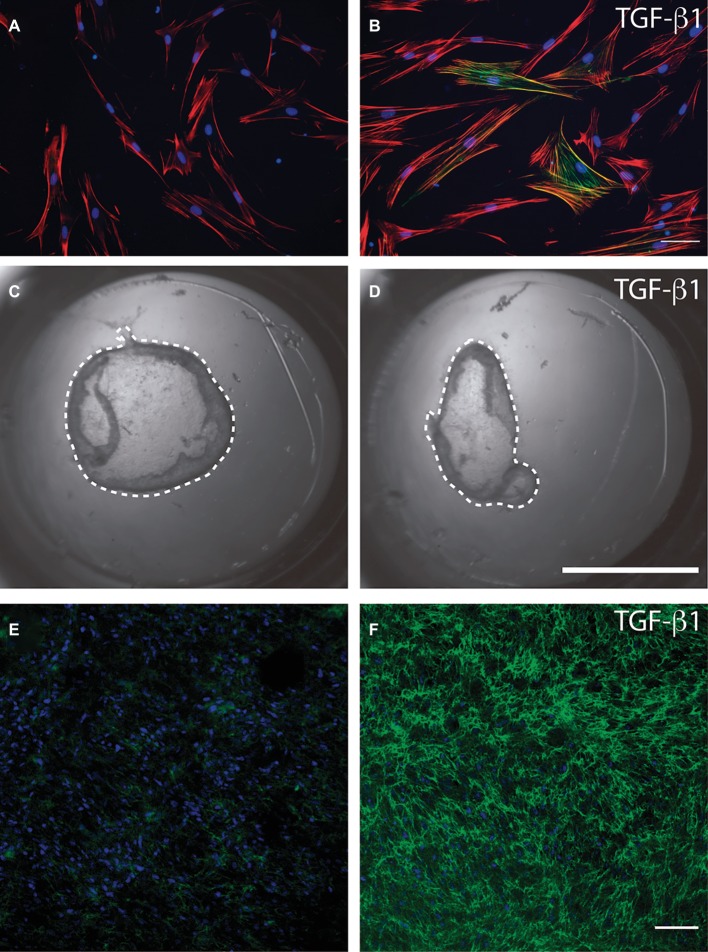
Methods to study myofibroblasts in cell culture. Serum-starved human gingival fibroblasts were cultured over a type I collagen film (50 mg/ml) and stimulated or not **(A)** with recombinant transforming growth factor beta 1 **(B)**. Immunofluorescence was used to identify the actin cytoskeleton (red), cell nuclei (blue), and alpha smooth muscle actin (green). Magnification bar equals 20 microns. Human gingival fibroblasts were cultured within a type I collagen gel (1 mg/ml) in the presence of 1% fetal bovine serum with the addition or not **(C)** of transforming growth factor beta 1 **(D)**. After 24 h collagen gels were released from the borders of the cell culture dish using a fine needle and the contraction of the gel was registered photographically. Dotted line represents the periphery of the collagen gel that must be compared to the periphery of the original gel area (area of the culture dish). Human gingival fibroblasts cultured within collagen gels were stimulated, or not, with TGF-b1 **(E** and **F)**. Collagen gels were stained for fibronectin (green) and cell nuclei (blue) and analyzed through confocal microscopy. Images show that TGF-b1 strongly stimulated fibronectin protein levels. Magnification bar = 40 μm. Images in this figure represent reanalysis of previously published data in Retamal et al., 2017.

Myofibroblasts are characterized by the *de novo* expression of alpha smooth muscle actin (a-SMA); they also exhibit reinforced adhesions to the extracellular matrix and an increased capacity to contract and remodel the extracellular matrix (Gabbiani et al., 1978; Desmoulière et al., 1993). Soluble factors that stimulate myofibroblast differentiation include heparin (Desmoulière et al., 1992) and biologically active transforming growth factor-b1 (TGF-b1) (Desmoulière et al., 1993). Myofibroblasts generate highly adhesive structures *in vivo* (the fibronexus) and *in vitro* (focal adhesions) that are enriched with the ED-A isoform of fibronectin, a specialized extracellular matrix protein that promotes cell attachment to collagen (Dugina et al., 2001). The phenotype and functions of fibroblasts and myofibroblasts have been examined using a wide variety of *in vitro* and *in vivo* methods. Figure 4 shows several experimental approaches used to study myofibroblastic differentiation, collagen remodeling, and fibronectin deposition. Figures 4A,B show the response of human gingival fibroblasts to TGF-b1, which, as previously indicated, stimulates myofibroblastic differentiation. Exposure of cells to TGF-b1 was associated with an expanded actin cytoskeleton (red) and with an increase in the protein levels of the myofibroblast marker a-SMA (green). TGF-b1 can also stimulate the contraction and remodeling of extracellular collagen. Figures 4C,D show collagen gels in which human gingival fibroblasts have been cultured. Treatment of collagen gels with TGF-b1 induced the contraction of collagen gels. Deposition of extracellular matrix components is an important function of myofibroblasts. Figures 4E,F show collagen gels populated with human gingival fibroblasts. Stimulation with TGF-b1 was associated with an increase in fibronectin as revealed by immunofluorescence (green).

As previously discussed, matrix stiffness is an important factor that modulates the synthetic, degradative, and remodeling activities of fibroblasts. Measurements of tissue stiffness made over time after wounding demonstrate a gradual increase, which may be attributed to the increased deposition and crosslinking of collagen (Ogawa, 2011; Chiron et al., 2012). Although increased stiffness may stimulate the differentiation of myofibroblasts necessary for normal wound healing, prolonged rigidity of the matrix may also promote scarring and fibrosis.

Secreted and membrane-anchored proteases expressed by fibroblasts play important roles in the maturation of granulation tissue. This effect has been observed in mice that are genetically deficient in matrix metalloproteinase-13 (MMP-13). These mice exhibit defective skin healing that is characterized by delayed granulation tissue formation and the appearance of myofibroblasts (Toriseva et al., 2012). Consistent with these findings, wound-induced granulation tissue does not mature normally in animals that are treated with MMP inhibitors (Mirastschijski et al., 2004). In addition to other possible explanations, these studies suggest that MMP-induced proteolysis mediates the release of matrix-bound components that regulate the activation/inactivation of growth factors and cytokines involved in granulation tissue development.

## Tissue Remodeling

During the phase of new tissue formation, the extracellular matrix is poorly organized and exhibits some of the features of connective tissues that are observed during fetal stages of development (Gurtner et al., 2008). This early stage wound healing matrix is enriched with hyaluronic acid, a nonsulfated, anionic glycosaminoglycan and contains increased levels of fibronectin, matricellular proteins like osteopontin and type III collagen (Beanes et al., 2002). In normal wound healing, the cells involved in the new tissue formation phase, which include myofibroblasts, macrophages, and endothelial cells, are deleted later on during the remodeling phase of wound healing (Desmoulière et al., 1995; Zhang et al., 2004). Specifically, myofibroblasts undergo apoptosis and are replaced by fibroblasts with a reduced capacity to secrete extracellular matrix components. During this phase, downregulation of the inflammatory response is also important for reducing the development of scar tissue (Mak et al., 2009); otherwise fibrotic lesions may develop.

The duration of the remodeling phase is highly variable and depends on several factors including the size of the wound and whether the injury has healed by primary or secondary intention. This phase starts at ~2 weeks after injury but may last for 1 year or more (Gurtner et al., 2008). During this phase, all of the critical biological responses activated after injury are downregulated and are gradually terminated. One of the important transformations detected during the tissue-remodeling phase is the substitution of the nascent extracellular matrix with a more mature and physically robust matrix that is gradually deposited into the wound. During the phase of wound healing when nascent matrix molecules are secreted, type III collagen is the main structural protein that is synthesized by fibroblasts. Over time, type III collagen is resorbed and is replaced by type I collagen fibers (Williams et al., 1984; Gurtner et al., 2008). Collagen fiber degradation, which is part of the collagen remodeling process, is mediated by members of the matrix metalloproteinase (MMP) family of proteinases. When sufficient amounts of cross-linked collagen fibrils have been secreted into the wound to provide sufficient wound strength that wound dehiscence does not occur, extensive collagen remodeling proceeds to optimize the tensile strength of wounds and to return tissues to their pre-wounded state (Gurtner et al., 2008).

Besides the degradation and synthesis of new collagen fibers, the extracellular matrix must be organized to restore the functional demands of the tissue. The reorganization of matrix structure is highly dependent on matrix receptors. Accordingly, fibroblasts adhere to collagen fibers through different types of adhesion receptors, which include the b1 integrin receptors and other adhesive proteins including the discoidin domain receptors (Staudinger et al., 2013). On their cytoplasmic domains, integrins are connected to actin filaments cytoskeleton through actin binding proteins like talin, filamin A, and paxillin, which contribute to the organization and signaling that is mediated through focal adhesions (Segal et al., 2001). These specialized adhesive organelles are also involved in the delivery of actomyosin generated tensile forces to mediate the condensation and alignment of collagen fibers in the extracellular matrix (Conrad et al., 1993).

The discoidin domain receptor 1 (DDR1) is a tyrosine kinase collagen adhesion receptor that mediates cell migration through association with nonmuscle myosin IIA (NMIIA). Recent data indicate that DDR1 interacts directly with NMIIA to enable collagen compaction by traction forces (Coelho et al., 2017). In this report, mechanical splinting of rat dermal wounds increased DDR1 expression and collagen alignment. Compared with wild type mice, in the periodontal ligament of DDR1 knockout mice, collagen reorganization was reduced >30%. *In vitro* studies from this report showed that tractional remodeling of collagen relied on DDR1 clustering, activation, and interaction of the DDR1 C-terminal kinase domain with NMIIA filaments. Collagen remodeling by tractional forces, DDR1 tyrosine phosphorylation, and myosin light chain phosphorylation were increased on stiff versus soft substrates. Thus, DDR1 clustering, activation, and interaction with NMIIA filaments enhance the collagen tractional remodeling that is important for collagen compaction that is important in wound healing and that may also contribute to tissue fibrosis (Coelho et al., 2017).

Through the integration of the adhesive activities of integrin and discoidin domain receptors, cell-mediated contraction enables the reorganization of collagen fibers, which will ultimately lead to the formation of a more physically robust and mature connective tissue. These biophysical properties of the fibrillar collagen-rich matrix are particularly important in the maintenance of periodontal attachment of teeth to alveolar bone and in the preservation of the integrity of the dentogingival junction.

Another important set of time-dependent modifications of the extracellular matrix during the remodeling phase is the gradually increased cross-linking of collagen fibers, which is mediated by several enzymes that include lysyl oxidases, lysyl hydroxylases, and transglutaminases that increase the stability and the tensile strength of the collagen network (Coelho and McCulloch, 2016).

## Experimental Approaches to Study Matrix Remodeling

The composition and structure of extracellular matrices are of critical importance for the development and the restoration of the structure and function of the normal periodontium. Because of the structural complexities and the remarkable load-bearing functions of the periodontal ligament and gingival connective tissues, the restoration of normal tissue function after clinical interventions is reliant on the tightly regulated synthesis and remodeling of fibrillar collagen matrices. Based on decades of experiments using cultured cells, animal models and in some cases human studies, a large array of methods has been developed to study reparative processes in periodontal tissues. As noted above, the structural and functional properties of collagen matrices vary widely and moreover depend to a large extent on the particular type of tissue (e.g., gingival lamina propria; periodontal ligament) and the state of health or disease of the tissue that is undergoing repair.

In health and disease, the particular extracellular matrix niche in which periodontal fibroblasts reside is associated with microenvironments with diverse biomechanical properties. These properties vary on a length scale of microns to millimeters. Biophysical approaches for modeling the mechanical properties of collagen matrices have indicated that the elasticity, topography, and roughness of fibrillar collagens strongly influences cell behavior, including spreading, migration, phagocytosis, and differentiation. Current thinking suggests that the ability of cells to mechanosense and to respond appropriately to the mechanical properties of the fibrillar collagen matrix is dependent in part on application of actomyosin-dependent contractile forces, as described above. For optimization of wound healing, it is helpful to understand the nature of the responses of periodontal fibroblasts to the mechanical properties of the matrix and how force-induced deformation of fibrillar collagen arrays can be measured.

One approach for assessing the nature of fibrillar collagen deformation fields uses naturally occurring matrix biopolymers (i.e., polymerized collagen), which are known to demonstrate more complicated mechanical behavior after application of forces than are simplified hydrogels (e.g., polyacrylamide-based gels). Notably, collagen gels can exhibit nonlinear viscoelastic behavior when subjected to cell-generated forces (see above), which may in turn promote strain stiffening of the collagen network and the formation of well-aligned arrays of collagen fibrils, as is seen in the periodontal ligament.

**Figure 5:**
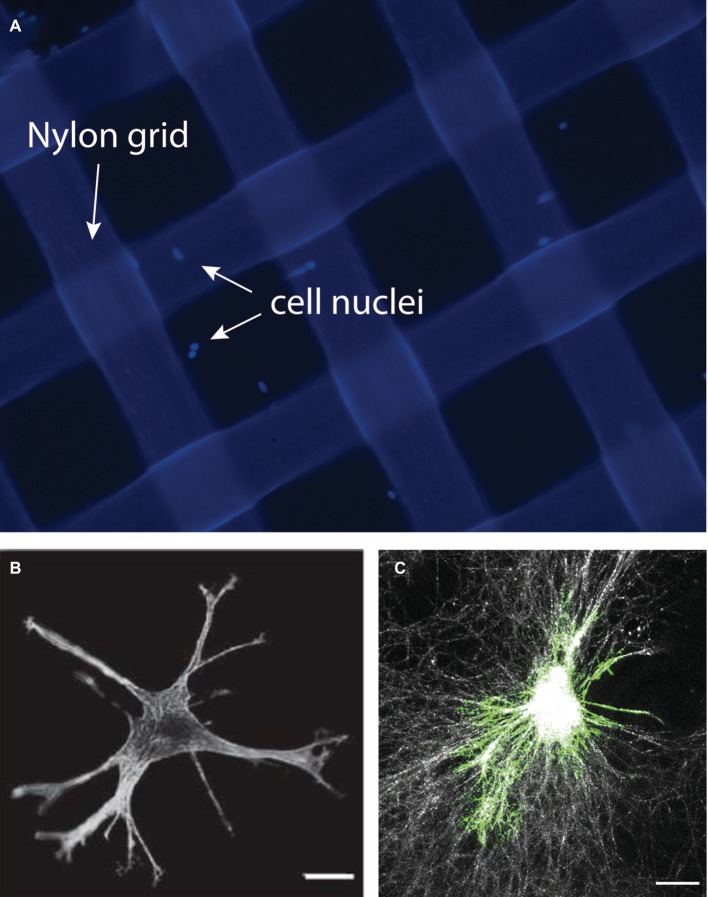
In this model, collagen-coated nylon grids of varying widths are created to assess the impact of lateral boundary sensing by periodontal fibroblasts. A collagen gel is prepared using the nylon grids. Image shows nylon grids (200 × 200 μm) treated with collagen. Cells were stained with DAPI to reveal the cell nuclei **(A)**. Cells cultured within the grid can be studied using immunofluorescence to identify cellular extensions **(B)** or to reveal collagen fiber organization and remodeling using the reflectance mode of the confocal microscope **(C)**. Images in this figure represent reanalysis of previously published data in Mohammadi et al., 2014.

Mechanosensing by collagen adhesion receptors (b1 integrins, discoidin domain receptors) in response to variations of the physical properties of fibrillar collagen arrays include the ability of these receptors to sense substrate roughness, topography, and the influence of lateral physical cues such as tissue boundary sensing in fibroblasts. This latter property may be particularly important in periodontal fibroblasts whose functions include the ability to “measure” periodontal ligament width and the distance between tissue boundaries (e.g., distance to tooth surface, thickness of lamina propria). Accordingly, we developed a model system to examine the ability of cells to remotely sense lateral boundaries. In this model system, floating, thin collagen gels are supported by rigid nylon grids of varying widths (Figure 5). Following the short-term spreading of cells on the floating collagen gel system, the dynamics, lengths, and numbers of cell extensions can readily be measured and related to the grid opening size. This latter property in turn determines the distance of cells from rigid physical boundaries. With the use of this system, we found that the generation of cell extensions in collagen gels (which is essential for collagen matrix remodeling) required expression of the β1 integrin, focal adhesion kinase, and actomyosin activity. The data arising from the use of this model indicate that the presence of physical boundaries interrupts the process of cell-mediated collagen compaction and fiber alignment in the collagen matrix and enhances the formation of cell extensions (Mohammadi et al., 2014). This cell culture platform could help researchers to define the roles of cell extensions and lateral mechanosensing on extracellular matrix remodeling by periodontal fibroblasts in the remodeling processes that are central to wound healing in these tissues.

## Conclusions

Fibroblasts play a critical role during periodontal wound healing. These cell populations are needed for the regeneration of a stable fibrillar connection between the tooth root, the gingiva, and the periodontal ligament. Importantly, regeneration of connective tissues involves different cellular activities driven by fibroblasts populations. These include the secretion of matrix molecules and the organization of these matrix components into functionally active fibers that finally restore the periodontium. Future studies should explore the cellular and molecular regulation of these cell populations and gain a more detailed understanding of the above-described mechanisms. This is critically important for the development of novel therapeutic approaches designed to regenerate periodontal tissues.

## Author Contributions

All authors listed have made a substantial, direct and intellectual contribution to the work, and approved it for publication.

### Conflict of Interest Statement

The authors declare that the research was conducted in the absence of any commercial or financial relationships that could be construed as a potential conflict of interest.
